# Concurrent Sagittal Craniosynostosis and Anterior Fontanelle Wormian Bone: Radiological Findings and Surgical Management in an Infant With Epispadias

**DOI:** 10.7759/cureus.95939

**Published:** 2025-11-02

**Authors:** Shrinivas Radder, Nivedita Radder

**Affiliations:** 1 Diagnostic Radiology/Pediatric Radiology, University of Arkansas for Medical Sciences, Arkansas Children's Hospital, Little Rock, USA; 2 Diagnostic Radiology, University of Arkansas for Medical Sciences, Little Rock, USA

**Keywords:** anterior fontanelle, computed tomography, pediatric imaging, sagittal craniosynostosis, skull deformity, wormian bone

## Abstract

Wormian bones are accessory ossicles that typically occur within cranial sutures. Their presence within fontanelles, particularly concurrent with craniosynostosis, represents an uncommon finding that poses unique diagnostic and management considerations. We present a one-month-old male infant born at 35 weeks of gestation, who presented with an abnormal skull configuration. Physical examination revealed scaphocephaly with a palpable sagittal ridge, bilateral temporal hollowing, and concurrent distal shaft epispadias. Computed tomography (CT) with three-dimensional (3D) reconstruction demonstrated sagittal craniosynostosis with an unusually large wormian bone within the anterior fontanelle. The patient underwent successful surgical management with extensive craniectomy, calvarial reconstruction using cranial distractor springs, and concurrent epispadias repair. The coexistence of craniosynostosis with wormian bone in the anterior fontanelle is rarely reported in the literature. This case highlights the importance of comprehensive imaging evaluation in infants with abnormal skull morphology and emphasizes the role of CT with 3D reconstruction in surgical planning. Anterior fontanelle wormian bones may occur in association with craniosynostosis, requiring careful radiological assessment and multidisciplinary surgical management. Three-dimensional CT reconstruction provides essential anatomical detail for operative planning in complex calvarial anomalies.

## Introduction

Wormian bones, also known as intrasutural or supernumerary bones, are accessory ossicles that form from independent ossification centers during fetal development [1]. While commonly found within cranial sutures, particularly the lambdoid suture, their occurrence within fontanelles is considerably less frequent [2]. The anterior fontanelle, located at the junction of the metopic, coronal, and sagittal sutures, typically remains patent until 12-18 months of age [3].

Craniosynostosis, the premature fusion of cranial sutures, affects approximately one in 2,500 live births, with sagittal synostosis being the most common form [4]. The concurrent presentation of craniosynostosis with anterior fontanelle wormian bone creates a unique clinical scenario that has been infrequently documented in the medical literature. This case report describes the radiological findings and successful surgical management of an infant presenting with this unusual combination of cranial anomalies, along with concurrent epispadias.

## Case presentation

A one-month-old male infant was referred to our pediatric clinic for evaluation of an abnormal skull shape noted by his parents. The child was born at 35 weeks of gestation to a primigravida mother following an uncomplicated pregnancy. The birth weight was 2,450 grams, and the Apgar scores were 8 and 9 at one and five minutes, respectively. The neonatal period was unremarkable, except for the skull abnormality and a genital anomaly identified at birth.

Clinical examination

Physical examination revealed a scaphocephaly skull configuration with a prominent palpable ridge along the sagittal suture. Bilateral temporal hollowing was evident, resulting from restricted lateral skull growth due to sagittal synostosis. The anterior fontanelle felt irregular on palpation, though no increased intracranial pressure signs were present. Neurological examination was age-appropriate, with normal primitive reflexes and symmetric movements.

Genital examination revealed distal shaft epispadias with a ventrally hooded prepuce. The urethral meatus was located on the dorsal aspect of the penile shaft, approximately 1.5 cm from the corona. Both testes were descended, and no other syndromic features or dysmorphisms were identified. Growth parameters were appropriate for gestational age, with head circumference tracking along the 75th percentile of the growth curve.

Imaging findings

**Figure 1:**
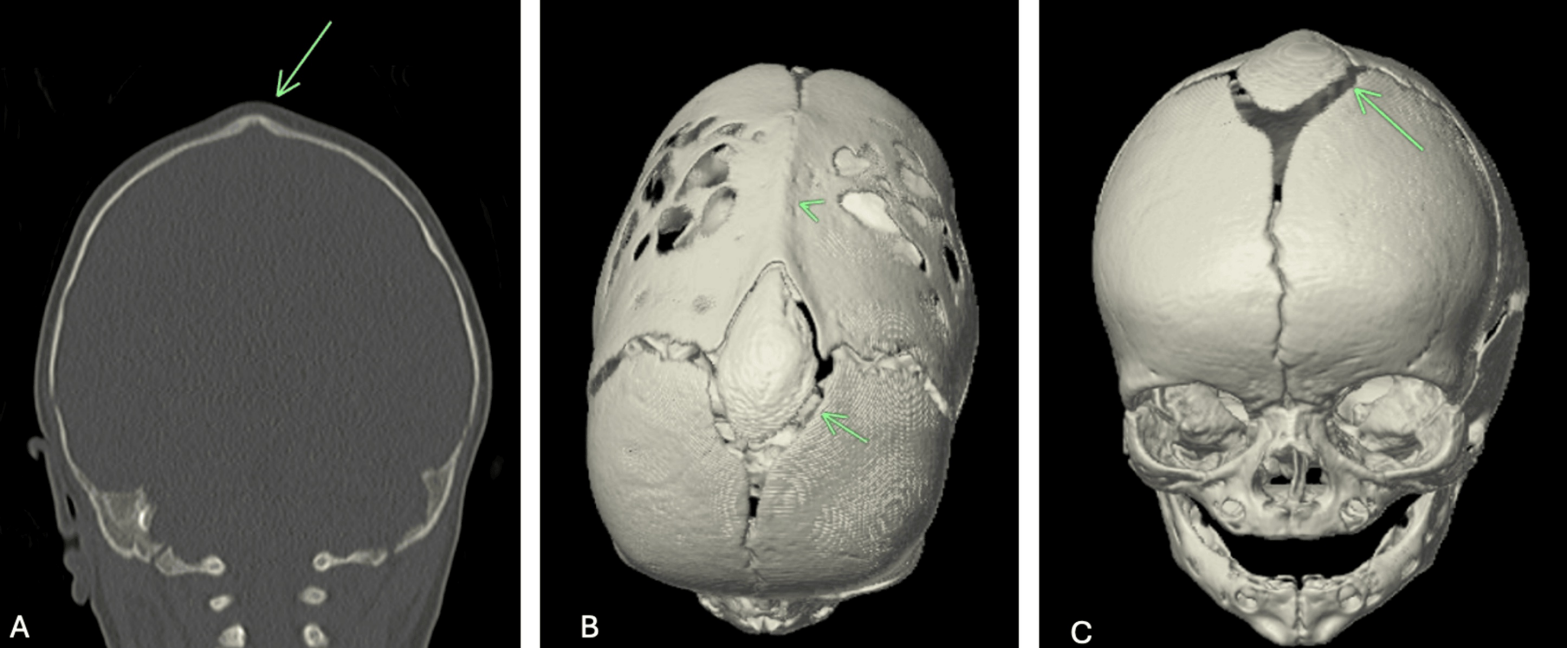
Three-dimensional computed tomography reconstruction of the skull demonstrating (A) complete fusion of the sagittal suture extending from the anterior to posterior aspect, presenting as an elevated osseous ridge indicative of sagittal craniosynostosis (arrow). (B) Axial view demonstrating a large, well-defined wormian bone measuring 4.3 × 2.5 cm occupying the anterior fontanelle region (arrow), with the fused sagittal suture visible posteriorly (arrowhead). (C) Coronal view highlighting the prominent anterior fontanelle wormian bone with intact cortical margins (arrow).

Non-contrast computed tomography of the head with three-dimensional reconstruction revealed complete osseous fusion along the entire length of the sagittal suture, manifesting as a prominent midline bony ridge consistent with sagittal craniosynostosis (Figures 1A, 1B). The skull demonstrated marked scaphocephaly, characterized by anterior-posterior elongation and biparietal narrowing, resulting in a cephalic index of 68%, which is significantly below the normal range of 75-85%. Within the expected location of the anterior fontanelle, a large, well-corticated accessory ossicle measuring 4.3 × 2.5 cm was identified, consistent with a wormian bone (Figures 1B, 1C). This anomalous bone displayed smooth, well-defined margins and standard trabecular architecture. Compensatory changes were evident with widening of the coronal and lambdoid sutures, likely secondary to the restricted growth pattern imposed by the sagittal fusion. The intracranial contents appeared normal without evidence of hydrocephalus, mass effect, or parenchymal abnormality. The three-dimensional reconstructions provided exceptional visualization of the complex spatial relationships between the synostosed sagittal suture, the anterior fontanelle wormian bone, and the patent surrounding sutures, proving invaluable for preoperative planning.

Management

Following multidisciplinary consultation involving pediatric neurosurgery, plastic surgery, and urology teams, the patient underwent surgical intervention at three months of age. The procedure included the following.

Cranial Surgery

An extensive strip craniectomy was performed along the fused sagittal suture. The large anterior fontanelle wormian bone was carefully elevated, preserving the underlying dura mater. Cranial distractor springs were placed to facilitate gradual calvarial expansion and remodeling. The wormian bone was repositioned and secured with absorbable sutures.

Urological Repair

Concurrent epispadias repair was performed using a modified Cantwell-Ransley technique with mobilization of the urethral plate and penile disassembly to achieve anatomical reconstruction.

Postoperative course

The patient tolerated both procedures well without complications. Serial postoperative head circumference measurements demonstrated normalization of growth, tracking along the 50th percentile compared to the preoperative 75th percentile, indicating successful cranial remodeling. Follow-up CT imaging at six months postoperatively showed excellent calvarial remodeling with a cephalic index of 76%. The cranial springs were removed at eight months of age as an outpatient procedure. The epispadias repair healed without complications, and the patient achieved normal urinary continence.

At 18 months follow-up, the child demonstrated normal neurodevelopmental milestones with an aesthetically satisfactory cranial shape. The anterior fontanelle had closed appropriately, incorporating the repositioned wormian bone into the normal cranial architecture.

## Discussion

This case presents a rare combination of sagittal craniosynostosis with a large anterior fontanelle and wormian bone, successfully managed with comprehensive surgical intervention. The concurrent presence of epispadias, while likely coincidental, added complexity to the surgical planning.

Wormian bones within fontanelles are considerably less common than those within sutures, with the reported incidence of anterior fontanelle wormian bones being particularly rare [5]. Previous studies have suggested that wormian bones may form in response to mechanical stress or altered tension forces during cranial development [6]. In the context of craniosynostosis, the altered biomechanical forces resulting from premature suture fusion may contribute to the formation of aberrant ossification centers.

The radiological evaluation of complex cranial anomalies has undergone significant evolution with the advent of 3D CT reconstruction. In our case, the 3D imaging provided crucial anatomical detail that facilitated surgical planning, particularly in determining the relationship between the wormian bone and the fused sagittal suture [7]. This imaging modality enabled a precise preoperative assessment, contributing to a successful surgical outcome.

The surgical management of craniosynostosis has evolved from simple suturectomy to more complex reconstructive procedures. The use of cranial distractor springs, as employed in this case, represents a dynamic approach that allows for gradual skull expansion and remodeling [8]. The presence of the anterior fontanelle wormian bone required careful surgical consideration to preserve its vascularity while achieving adequate decompression and aesthetic reconstruction.

While isolated wormian bones are often considered benign variants, their association with craniosynostosis warrants careful evaluation. Previous reports have described wormian bones in various syndromic craniosynostoses; however, isolated sagittal synostosis with an anterior fontanelle wormian bone remains rarely documented [9]. Our case did not exhibit features suggestive of an underlying syndrome, and genetic testing was not pursued due to the absence of other dysmorphic features or a family history.

The concurrent epispadias in our patient appears to be an isolated finding rather than part of a syndromic presentation. Epispadias, occurring in approximately one in 117,000 male births, represents a rare congenital anomaly of the genitourinary system. The successful simultaneous surgical correction of both anomalies reduced the need for multiple anesthetic exposures and hospitalization episodes, improving overall patient care efficiency [10].

## Conclusions

This case demonstrates the successful diagnosis and management of a rare combination of sagittal craniosynostosis with anterior fontanelle wormian bone in an infant. Three-dimensional CT reconstruction proved invaluable for surgical planning, and the use of cranial distractor springs facilitated excellent calvarial remodeling. The concurrent repair of epispadias highlights the benefits of coordinated multidisciplinary surgical care. Pediatric radiologists should be aware of this unusual presentation, as early recognition and appropriate imaging can optimize surgical outcomes.
